# The accuracy of symptoms, signs and diagnostic tests in the diagnosis of left ventricular dysfunction in primary care: A diagnostic accuracy systematic review

**DOI:** 10.1186/1471-2296-9-56

**Published:** 2008-10-08

**Authors:** V Madhok, G Falk, A Rogers, AD Struthers, FM Sullivan, T Fahey

**Affiliations:** 1 Tayside Centre for General Practice, Division of Community Health Sciences, University of Dundee, Mackenzie Building, Dundee DD2 4BF, UK; 2 Department of General Practice, Royal College of Surgeons in Ireland, 120 St. Stephen's Green, Dublin 2, Ireland; 3 Division of Medicine and Therapeutics, University of Dundee, Ninewells Hospital, Dundee DD1 9SY, UK

## Abstract

**Background:**

To assess the accuracy of findings from the clinical history, symptoms, signs and diagnostic tests (ECG, CXR and natriuretic peptides) in relation to the diagnosis of left ventricular systolic dysfunction (LVSD) in a primary care setting.

**Methods:**

Diagnostic accuracy systematic review, we searched Medline (1966 to March 2008), EMBASE (1988 to March 2008), Central, Cochrane and ZETOC using a diagnostic accuracy search filter. We included cross-sectional or cohort studies that assess the diagnostic utility of clinical history, symptoms, signs and diagnostic tests, against a reference standard of echocardiography. We calculated pooled positive and negative likelihood ratios and assessed heterogeneity using the I^2 ^index.

**Results:**

24 studies incorporating 10,710 patients were included. The median prevalence of LVSD was 29.9% (inter-quartile range 14% to 37%). No item from the clinical history or symptoms provided sufficient diagnostic information to "rule in" or "rule out" LVSD. Displaced apex beat shows a convincing diagnostic effect with a pooled positive likelihood ratio of 16.0 (8.2–30.9) but this finding occurs infrequently in patients. ECG was the most widely studied diagnostic test, the negative likelihood ratio ranging from 0.06 to 0.6. Natriuretic peptide results were strongly heterogeneous, with negative likelihood ratios ranging from 0.02 to 0.80.

**Conclusion:**

Findings from the clinical history and examination are insufficient to "rule in" or "rule out" a diagnosis of LVSD in primary care settings. BNP and ECG measurement appear to have similar diagnostic utility and are most useful in "ruling out" LVSD with a normal test result when the probability of LVSD is in the intermediate range.

## Background

Left Ventricular Systolic Dysfunction (LVSD) is a major clinical problem worldwide. In the UK alone it has been estimated that 878,000 people have definite or probable LVSD, with 63,000 new cases annually [1]. A diagnosis of LVSD carries a poor prognosis with 40% of patients dying within a year of first diagnosis. People living with LVSD report a significantly lower quality of life than the general population. The annual cost to the National Health Service attributable to LVSD has been estimated at around £625 million [1].

The European Society of Cardiology (ESC) state that there is no exclusive definition of Heart Failure although it is recognised as a syndrome in which abnormal cardiac function is a cause for the heart being unable to pump blood at the rate required to meet the needs of the metabolizing tissues [2]. LVSD is one possible reason for heat failure characterised by compromised ventricular function leading to a variety of symptoms such as fatigue, breathlessness, and oedema. The ESC has proposed that the diagnosis of LVSD depends on the presence of some or all of these symptoms along with objective evidence of cardiac dysfunction at rest [2]. Accurate diagnosis of LVSD with echocardiography is important for two reasons: firstly to determine the underlying cause of heart failure-broadly LVSD, valve disease, or diastolic dysfunction of the left ventricle (heart failure with preserved systolic function); and secondly to initiate treatments to alleviate symptoms, delay progression and improve prognosis [3].

National and international guidelines on the diagnosis and management of LVSD have been published, [2-4] and current diagnostic algorithms support a structured pathway of history, examination, and diagnostic testing with brain natriuretic peptide (BNP) and electrocardiogram (ECG) as part of the initial diagnostic assessment by family physicians (GPs) [3]. As access to echocardiography services is limited by the availability of equipment and appropriately trained personnel, this approach is likely to remain the most cost effective way of diagnosing LVSD in the community [5].

Previous reviews have sought to assess the diagnostic test accuracy of BNP or ECG measurement in primary care or the accident and emergency environments [6-14]. However, these studies assessed BNP and ECG testing in isolation; not in the context of initial presentation (symptom and signs) and the incremental diagnostic value of BNP or ECG measurement for risk-stratifying patients in terms of onward referral for echocardiography.

The aim of this systematic review and meta-analysis is to identify diagnostic accuracy studies based in primary care that evaluate both individual symptoms and signs, BNP and ECG measurement in patients presenting with suspected LVSD, as a means of evaluating and quantifying diagnostic algorithms for suspected LVSD in primary care.

## Methods

We followed the recommended methods of the Cochrane Diagnostic Accuracy Group for systematic reviews of Diagnostic tests [15]. A search strategy was devised taking into account existing filters that identify diagnostic accuracy studies (Additional file 1) [16]. The following were searched: MEDLINE (1966 to March 2008), EMBASE (1988 to March 2008), CENTRAL, and ZETOC (conference proceedings). Additionally, AR searched the bibliographies of all relevant retrieved papers. No restrictions were placed on language of publication or publication status.

### Study Selection

Inclusion criteria were as follows:

• Population: study participants must have been recruited from a community or primary care setting and have symptoms suggestive of LVSD. Screening studies in asymptomatic patients were excluded as were case control studies in which control patients were compared with patients with previously established LVSD.

• Study design and reference standard: studies should assess diagnostic accuracy by means of cross sectional study and application of echocardiography as the reference standard test.

• Index tests: studies must assess the value of symptoms, signs, ECG, Chest X ray (CXR) and/or natriuretic peptides in diagnosing LVSD.

• Outcome measures: studies must report data that will allow 2 × 2 table construction for the assessment of diagnostic accuracy for individual symptoms, signs or diagnostic tests.

Three reviewers (VM, AR, and GF) independently reviewed the titles and/or abstracts of retrieved citations. The full text of potentially relevant studies were retrieved and reviewed. Disagreements were resolved by consensus or by consultation with a fourth reviewer (TF). We independently assessed the quality of each study and extracted data to construct a 2 × 2 table for each symptom, sign or diagnostic test.

### Assessment of Study Quality

The QUADAS quality assessment tool was modified alongside a quality assessment tool for clinical prediction rules to produce a seven-point quality assessment score for each included study [17,18]. Any disagreement on study quality was resolved by consensus with a third reviewer (TF).

### Data Extraction

Two independent reviewers extracted data onto a spreadsheet for the construction of 2 × 2 tables.

### Quantitative data synthesis

Meta-DiSc 1.2 software was used to analyse the extracted data [19]. We calculated positive and negative likelihood ratios for each study, with random methods used in the analysis. Heterogeneity was assessed using the I^2 ^index [20], where I^2 ^≤ 50% studies were considered to be sufficiently homogeneous to allow pooling and produce a summary estimate of diagnostic test accuracy. In situations of significant between-study heterogeneity the range of positive and negative likelihood ratios are presented [21].

## Results

**Figure 1:**
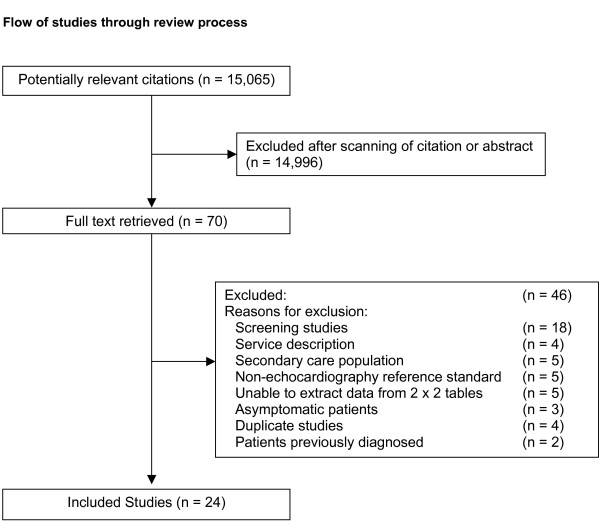
Flow of studies through review process.

We identified 15,065 potentially relevant articles. After scanning titles and abstracts, we retrieved the full text of 70 articles for further evaluation. Twenty-four studies met our inclusion criteria and were included in the final analysis (Figure 1) [22-45]. Reasons for exclusion of studies (n = 46) included, incorrect study design, population derived from duplicate publication, and population included patients previously diagnosed with LVSD.

### Characteristics of studies

All included studies were cross-sectional in design and were conducted in a primary or ambulatory care setting (Additional file 2). Of the 10,710 patients with symptoms or signs of LVSD, the median prevalence of LVSD was 29.9% (inter-quartile range 14% to 37%). One study also included patients considered to be at high risk of LVSD on the basis of previous clinical history [42]. None of the studies included patients with previous echocardiographic diagnosis of LVSD. Of the included studies, five assessed the diagnostic usefulness of various symptoms and signs, ten assessed ECGs and fourteen assessed one or more of the natriuretic peptides.

### Quality of included studies

The overall quality of included studies is summarized in Additional file 3. Adequate description and inclusion of important predictors (symptoms, signs and diagnostic tests) occurred in half of the included studies. Similarly absence of blinding between index test (symptom, sign or diagnostic test) and reference standard test occurred in half of the included studies.

### Definition of predictors of LVSD

A variety of definitions of ECG abnormality were used. In two studies, ECGs were interpreted by GPs, and in the remaining studies interpretation was by secondary care physicians. Four studies did not explicitly describe criteria for deciding whether an ECG was abnormal. The remainder used a variable number of features such as evidence of previous MI, left ventricular hypertrophy and bundle branch block. One of the studies assessing chest x ray used only a cardiothoracic ratio greater than 0.5 as an indicator of abnormality. The other study used the presence of either pulmonary oedema or cardiomegaly, neither of which was objectively quantified. The cut-off points for natriuretic peptides had wide variability, and were chosen for differing reasons. In some studies the test manufacturer's recommended cut-off was used, and in others it was chosen to give optimum test performance in their study. BNP cut-offs varied from 10 pg/mL (2.89 pmol/L) to 100 pg/mL (28.9 pmol/L) (median 38.51 pg/mL (11.13 pmol/L)). Where studies reported results for more than one cut-off point, we included only the cut-off closest to the median. NTproBNP cut-offs ranged from 92.77 pg/mL (10.97 pmol/L) to 449.07 pg/mL (53.1 pmol/L) (median 146.73 pg/mL (17.35 pmol/L)). Where multiple cut points were reported we used only the cut-off closest to the median. The two studies assessing NTproANP chose cut-offs of 537.6 pmol/L and 0.8 nmol/L.

### Definition of reference standard test

All studies used a reference standard of echocardiography. In four studies the Echocardiogram was assessed on a purely qualitative basis. Four other studies used the European Society of Cardiology (ESC) guidelines for LVSD, which combine symptoms of LVSD with objective evidence of cardiac dysfunction at rest [2]. The most commonly reported quantitative measurement of cardiac function was ejection fraction. Twelve studies explicitly used ejection fraction as a measure of cardiac function with cut-offs ranging from 35% to 50% (median 40%). The ESC guidelines suggest that an ejection fraction < 40%, implies abnormal systolic function [2].

### Diagnostic value of symptoms, signs and diagnostic tests

The diagnostic value from the clinical history (four items), symptom (four items), clinical signs (five items) and available diagnostic tests are summarized in Additional file 4. No item from the clinical history or symptoms provided sufficient diagnostic information to rule in or rule out LVSD. Of the clinical signs assessed, displaced apex beat shows a convincing diagnostic effect with a pooled positive likelihood ratio of 16.0 (8.2–30.9), but this was based on findings from only two studies. The presence of a third heart sound shows a wide range of positive likelihood ratios (1.6 to 32.4) as does the presence of a raised jugular venous pulse (2.7 to 7.4) The range of positive likelihood ratios for peripheral oedema (0.96 to 1.48) suggests that this is an uninformative clinical sign (Additional file 4)

In terms of diagnostic tests, chest radiography appears to be uninformative with positive likelihood ratios in two studies being 1.2 and 1.7. ECG was the most widely studied diagnostic test, the negative likelihood ratio ranging from 0.06 to 0.76 (Additional file 4). Two studies did report on the combination of ECG and a positive history of myocardial infarction with a pooled positive likelihood ratio of 2.8.

Not unexpectedly, given the wide variation in diagnostic threshold, BNP results were strongly heterogeneous, with negative likelihood ratios ranging from 0.02 to 0.80. Similarly, NTproANP and NTproBNP both had high heterogeneity with a broad range of reported negative likelihood ratios (Additional file 4).

## Discussion

This diagnostic accuracy systematic review of LVSD shows that the diagnostic value of single items from the clinical history or symptoms in patients presenting to their family practitioner is modest. Likelihood ratios for some clinical signs-raised JVP, displaced apex beat and third heart sound, appear to be more diagnostically useful in ruling in LVSD but are based on a small number of studies in which the prevalence of these clinical signs is low. It is likely these florid clinical signs occur in patients for whom there is little diagnostic uncertainty concerning LVSD compared to the more typical range of patients whose diagnosis needs to be established in routine clinical practice in primary care. Patients with a history of prior MI are likely to be a more diagnostically useful group in terms of probability of LVSD (Additional file 4). These findings highlight the challenges of evaluating the diagnostic utility of items from clinical history and examination in primary care. "Ruling in" or "ruling out" a diagnosis of LVSD on clinical grounds is not likely to be possible. Insufficient evidence exists to determine whether combinations of signs and symptoms are more diagnostically useful.

**Figure 2:**
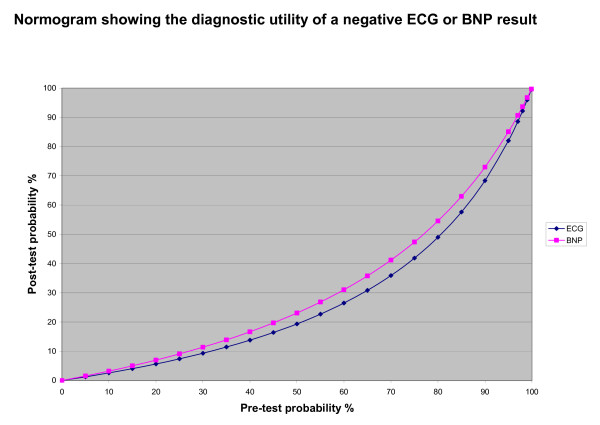
Normogram showing diagnostic utility.

Addition of near patient diagnostic tests does have value in terms of "ruling out" the probability of LVSD. ECG and BNP testing both appear to provide important diagnostic information when they are negative in "ruling out" the presence of LVSD. The nomogram in Figure 2 quantifies the importance of these diagnostic tests in the context of the prior probability/prevalence of LVSD. In situations where the prior prevalence of LVSD is 30%, a figure that matches the median prevalence in the included studies in this diagnostic accuracy review, a negative ECG or BNP lowers the post-test probability of LVSD to 10%. In these situations, pursuit of alternative diagnosis or a watchful waiting (rather than referral) strategy would seem to be a cost effective and clinically appropriate approach [46].

The results of this review are consistent with previously published research in relation to diagnostic testing for LVSD in terms of BNP measurement [10-12,14]. This review shows that the diagnostic value of ECG measurement appears to be equivalent to BNP – most useful as a "ruling out" test in situations of low to intermediate pre-test probability (Figure 2). Furthermore, including the diagnostic values of clinical history findings, symptoms and signs more accurately reflects the diagnostic approach of clinical practice, where the probability of the target disorder – in this case LVSD – is revised with additional information from the clinical history, along with the presence or absence of symptoms and signs in an individual patient [47].

The significant heterogeneity between many of the included studies is not unusual in terms of diagnostic accuracy systematic reviews [47], reflecting differences in the definitions used in the reference standard echocardiograms in relation to LVSD, different cut-points for natriuretic peptide measurement and differences in the definition of "abnormal" ECG findings (Additional file 2). Despite these shortcomings, a consistent finding is that the likelihood ratios for individual features from the clinical history and examination are modest, and are not likely to produce definitive revisions in the probability of LVSD alone. The findings from this systematic review also support the sequential diagnostic algorithm recommended in clinical guidelines for heart failure [4,48]. Our findings would also support a more quantified approach to the diagnosis of LVSD, which could be incorporated into a computer-based clinical decision support system [49].

Future studies will need to address the incremental value of combined findings from the clinical history and examination. The relative value of BNP or ECG measurement is likely to relate to issues of experience and training of family doctors in ECG interpretation, which can be variable [5,50]. Future studies should also incorporate cost effectiveness data in relation to alternative diagnostic testing, particularly in relation to determining other structural abnormalities such as valvular disease and left ventricular hypertrophy [51]. Lastly, as evidence accumulates in relation to the strong prognostic role of natriuretic peptides in patients with heart failure [52,53], questions about the definition and value of the diagnostic label of heart failure has arisen using echocardiography as a reference standard [54]. However, the randomised trial evidence for therapeutic interventions are based on studies that use echocardiographic left ventricular ejection fraction as an entry criteria [55]. Future diagnostic accuracy studies may wish to consider use of supplementary or alternative reference standards in conjunction with echocardiography and follow up patients to assess their longer term outcome [53].

## Conclusion

In conclusion, this diagnostic accuracy systematic review shows that findings from the clinical history and examination are insufficient to "rule in" or "rule out" a diagnosis of LVSD. BNP and ECG measurement appear to have similar diagnostic utility and are most useful in "ruling out" LVSD with a normal test result when the probability of LVSD is in the intermediate range. Future studies should assess the combined value of clinical findings and diagnostic testing in primary care.

## Competing interests

ADS is a consultant to Stirling Medical who manufacture diagnostic BNP products. All other authors declare no conflict of interest.

## Authors' contributions

VM, GF and AR all contributed to searching for studies, summarizing included studies and extracting relevant data. VM and GF were responsible for data analysis supported by TF, ADS and FMS. TF, ADS and FMS conceived and designed the study. AR, VM, GF and TF helped to draft the manuscript. All authors read and approved the final manuscript.

## Pre-publication history

The pre-publication history for this paper can be accessed here:



## Supplementary Material

Additional file 1 **Table 1.** Search Strategy Click here for file

Additional file 2 **Table 2.** Characteristics of study. Click here for file

Additional file 3 **Table 3.** Methodological standards for studies. Click here for file

Additional file 4 **Table 4.** Clinical values Click here for file
