# Supplementary material for: The accuracy of symptoms, signs and diagnostic tests in the diagnosis of left ventricular dysfunction in primary care: A diagnostic accuracy systematic review
Source: BMC Fam Pract. 2008 Oct 8;9:56. doi: 10.1186/1471-2296-9-56 (PMC2569936; doi:10.1186/1471-2296-9-56)
Supplement: Additional file 1 — Table 1. Search Strategy [file 1471-2296-9-56-S1.doc]

| Search Strategy |
| --- |
| 1. exp heart failure, congestive/ |
| 2. exp ventricular dysfunction/ |
| 3. heart failure.tw. |
| 4. cardiac failure.tw. |
| 5. (ventricular adj3 dysfunction$).tw. |
| 6. cardiac dysfunction.tw. |
| 7. cardiac insufficiency.tw. |
| 8. exp ventricular function/ |
| 9. cardiac function.tw. |
| 10. ventricular function.tw. |
| 11. myocard$ dysfunction$.tw. |
| 12. systolic dysfunction.tw. |
| 13. heart insufficiency.tw. |
| 14. cardiac insufficiency.tw. |
| 15. cardiac overload.tw. |
| 16. cardiac output, low/ |
| 17. chf.tw. |
| 18. ccf.tw. |
| 19. hf.tw. |
| 20. lvsd.tw. |
| 21. or/1-20 |
| 22. radiography, thoracic/ |
| 23. chest x-ray$.tw. |
| 24. chest radiograph$.tw. |
| 25. exp echocardiography/ |
| 26. echocardiograph$.tw. |
| 27. electrocardiography/ |
| 28. electrocardiograph$.tw. |
| 29. ecg.tw. |
| 30. physical examination/ |
| 31. medical history taking/ |
| 32. exp auscultation/ |
| 33. exp heart auscultation/ |
| 34. (clinical adj (exam or exams or examination$)).tw. |
| 35. (physical adj (exam or exams or examination$)).tw. |
| 36. clinical evaluation$.tw. |
| 37. clinical assessment$.tw. |
| 38. auscultation.tw. |
| 39. "signs and symptoms"/ |
| 40. "signs and symptoms".tw. |
| 41. exp natriuretic peptides/ |
| 42. natriuretic peptide$.tw. |
| 43. bnp.tw. |
| 44. anp.tw. |
| 45. or/22-44 |
| 46. 21 and 45 |
| 47. exp "sensitivity and specificity"/ |
| 48. exp heart failure, congestive/di |
| 49. sensitivity.tw. |
| 50. specificity.tw. |
| 51. false positive$.tw. |
| 52. false negative$.tw. |
| 53. reference values/ |
| 54. Mass screening/ |
| 55. screening.tw. |
| 56. reference standards/ |
| 57. predictive value$.tw. |
| 58. predictive model$.tw. |
| 59. gold standard$.tw. |
| 60. exp diagnostic errors/ |
| 61. diagnosis, differential/ |
| 62. reference value$.tw. |
| 63. likelihood ratio$.tw. |
| 64. likelihood ratio$.tw. |
| 65. exp ventricular dysfunction/di |
| 66. di.fs. |
| 67. or/47-66 |
| 68. 46 and 67 |
| 69. exp animals/ not human/ |
| 70. 68 not 69 |
