# Supplementary material for: The accuracy of symptoms, signs and diagnostic tests in the diagnosis of left ventricular dysfunction in primary care: A diagnostic accuracy systematic review
Source: BMC Fam Pract. 2008 Oct 8;9:56. doi: 10.1186/1471-2296-9-56 (PMC2569936; doi:10.1186/1471-2296-9-56)
Supplement: Additional file 2 — Table 2. Characteristics of study. [file 1471-2296-9-56-S2.doc]

##### Characteristics of studies of symptoms, signs and tests when diagnosing LVSD in community patients

| Author  Year  Number of patients | Prior LVSD | Prevalence of symptoms/signs | Patient population | Diagnostic tests assessed  Cut points | Gold  Standard  Definition | Diagnostic utility  Positive likelihood ratio (LR) or Odds ratio (OR) | Notes |
| --- | --- | --- | --- | --- | --- | --- | --- |
| Alehagen et al [22]  2003  N=415 | 13% | 415 elderly patients presenting to a primary care centre with symptoms suggestive of HF | Not reported | NT-proBNP  NT-ANP  NT-proBNP ≥138pg/mL (40pmol/L)  NT-ANP ≥ 0.8nmol/L  (= 800pmol/L) | ECHO  EF<40% | | NT proBNP cutoff | LR | | --- | --- | | ≥ 138.40pg/mL (40pmol/L) | 1.1 | |  |
| Aspromonte et al [23]  2006  N=253 | 62.1% | 253 GP-referred patients with symptoms of HF aimed at the early diagnosis of mild HF | Symptoms – not reported   | Signs | % | | --- | --- | | IHD | 32% | | Hypertension | 41% | | AF | 17% | | Renal Failure | 6% | | COPD | 9% | | Diabetes Mellitus | 11% | | LBBB | 15% | | BNP  ≥ 30pg/mL (8.7pmol/L)§  ≥ 50pg/mL (14.5pmol/L)  ≥ 80pg/mL 23.1pmol/L)  ≥ 100pg/mL (28.9pmol/L) | ECHO  EF<45% | | BNP cutoff | LR | | --- | --- | | ≥ 30pg/mL (8.7pmol/L) | 40.2 | | Data for different cut-points of BNP obtained from author, 30ng/L cut-point used |
| Atisha et al [24]  2004  N=202 | 48.5% | 202 patients presenting with symptoms suggestive of HF | Signs – Not reported   | Symptoms | % | | --- | --- | | Stop for breath while walking at own pace | 30% | | Dyspnoea after 100 yards of walking | 35% | | Orthopnoea | 21% | | PND | 23% | | Night cough | 23% | | Fatigue | 40% | | Weakness | 36% | | Oedema | 40% | | BNP  BNP ≥ 20pg/mL (5.8pmol/L) | ECHO  EF<50%, valvular disease, hypertrophy, abnormal end-diastolic volumes | | BNP cutoff | LR | | --- | --- | | ≥ 69.2pg/mL (20pmol/L) | 1.4 | |  |
| Cowie et al [25]  1997  N=122 | 29% | Consecutive patients referred from primary care with provisional diagnosis to rapid-access clinic | Not reported | ANP  BNP  ANP ≥ (18.1pmol/L)  BNP ≥ 76.8pg/mL (22.2pmol/L) | ECHO  European Society of cardiology* | | Test | LR | OR | | --- | --- | --- | | ANP | 3.5 | 8.4 | | BNP | 6.1 | 2.3 | | Diagnostic utility of S&S not reported.  BNP strongest predictor |
| Davie et al [26]  1996  N=534 | 18% | Patients referred to an open access ECHO hospital clinic | Not reported | ECHO  Abnormalities were: AF, previous MI, LVH, BBB or LAD | ECHO  When possible LVSD was quantified in terms of FS derived from M mode; otherwise assessed simply as preserved or impaired | | Test | LR | | --- | --- | | Abnormal ECG | 2.4 | | “Minor abnormalities” included- atrial enlargement, brady/tachycardia, broad QRS, poor R wave progression, RAD, first degree AV block, non-specific ST change |
| Davie et al [27]  1997  N=259 | 16% | Consecutive patients referred from primary care with provisional diagnosis to rapid-access clinic | |  | % | | --- | --- | | MI | 20% | | Hypertension | 33% | | Diabetes | 3% | | Exertional dyspnoea | 86% | | Orthopnoea | 24% | | PND | 22% | | Oedema | 52% | | Pulse >100 | 10% | | Raised JVP | 5% | | Gallop | 5% | | Murmur | 36% | | Crackles | 24% | | Oedema | 15% | | Displaced apex | 14% | | History & Examination  Not relevant | ECHO  FS <25% or when not possible to quantify “qualitatively assessed as either preserved of significantly impaired” | |  | LR | | --- | --- | | MI | 4.21 | | Hypertension | 0.6 | | Diabetes | 6.0 | | Exertional dyspnoea | 1.2 | | Orthopnoea | 0.8 | | PND | 2.0 | | Oedema | 0.9 | | Pulse >100bpm | 2.8 | | Raised JVP | 8.5 | | Gallop | 24 | | Murmur | 1.5 | | Crackles | 1.3 | | Oedema | 1.4 | | Displaced apex | 16.5 | | Unadjusted combinations of S&S also presented with combination of past MI & displaced apex beat LR 99 |
| Fahey et al [28]  2007  N=458 | 8% | GP referrals - patients with suspected LVSD | |  | % | | --- | --- | | Dyspnoea | 87% | | Oedema | 66% | | Tiredness | 56% | | Orthopnoea | 10% | | PND | 13% | | Wheeze | 38% | | Cough | 44% | | Chest discomfort | 30% | | MI | 19% | | Hypertension | 48% | | Diabetes | 12% | | Overweight | 79% | | AF | 10% | | Elevated JVP | 6% | | Displaced Apex | 1% | | Murmur | 21% | | Chest crepitations | 18% | | Chest Rhonchi | 15% | | Lower limb oedema | 30% | | ECHO  Any abnormality from normal (defined in paper) | ECHO  EF<50%, FS <25%, visual assessment | |  | LR | | --- | --- | | Dyspnoea | 1.1 | | Oedema | 0.9 | | Tiredness | 0.9 | | Orthopnoea | 3.6 | | PND | 0.3 | | Wheeze | 1.5 | | Cough | 1.2 | | Chest discomfort | 1.7 | | MI | 3.1 | | Hypertension | 0.6 | | Diabetes | 0.9 | | Overweight | 0.7 | | AF | 1.9 | | Elevated JVP | 12.5 | | Displaced Apex | 8.9 | | Chest crepitations | 0.4 | | Lower limb oedema | 1.1 | | ECG | 1.9 | |  |
| Fox et al [29]  2000  N=383 | 26% | Patients referred from primary care with provisional diagnosis to rapid-access clinic | |  | % | | --- | --- | | MI | 11% | | Hypertension | 39% | | Diabetes | 10% | | Exertional dyspnoea | 36% | | Orthopnoea | 24% | | Ankle oedema | 54% | | History & Examination  ECG & CXR  Abnormal result | ECHO  European Society of cardiology* | | Sign | LR | | --- | --- | | Oedema | 1.5 | | Lung crepitations | 2.0 | | Normal ECG & CXR rule out Dx of HF  All S&S not reported |
| Fuat et al [30]  2006  N=297 | 38.4% | Patients referred by GPs with symptoms & signs suggestive of HF to diagnostic clinics at two hospitals | |  | % | | --- | --- | | Hypertension | 27% | | IHD | 33% | | Previous MI | 24% | | ECG  BNP  NTproBNP  ECG: Minnesota criteria  BNP >40pg/mL (11.6pmol/L)  NTproBNP >150pg/mL  (17.7pmol/L) | ECHO  Visual assessment, EF & wall motion index. | | Test | LR | | --- | --- | | ECG | 1.9 | | BNP | 1.5 | | NTproBNP | 1.6 | |  |
| Gustafsson et al [31]  2003  N | 9% | Primary-care patients with a provisional diagnosis of HF referred by GPs for ECHO at Copenhagen Family physician’s Laboratory | |  | % | | --- | --- | | History of IHD | 13% | | Hypertension | 19% | | Diabetes | 2% | | NT-proBNP  450pg/mL (53.1pmol/L)  (>75 years)  93pg/mL (11.0pmol/L)  (Male)  144pg/mL (17.0pmol/L)  (Female) | ECHO  LVEF estimated using:  1) Motion wall score index if regional wall motion abnormalities.  2) FS based on M-mode scans of long-axis parasternal view if homogenous regional wall motion.   | LVSD | LVEF | | --- | --- | | Normal | > 0.40 | | Moderate | < 0.40 | | Severe | < 0.30 | | NT-proBNP |  |
| Hess et al [32]  2005  N=473 | 24% | Patients referred to cardiologists for assessment of cardiac state | |  | % | | --- | --- | | Dyspnoea | 44% | | Oedema | 10% | | History of MI | 16% | | HF medication | 75% | | NT-proBNP  >125 pg/mL (=14.75pmol/L) | ECHO  LVEF classified into 3 groups:  EF>30%  EF 30-50%  EF >50% | | NT proBNP cutoff | | --- | | >125 pg/mL | |  |
| Hobbs et al [33]  2002  N=591 | 9% | Subset of population study, patients with HF, taking diuretics or at high risk of HF | |  | % | | --- | --- | | MI | 14.7% | | Angina | 21.5% | | Hypertension | 39.3% | | Diabetes | 11.5% | | NT-proBNP  >304.45pg/mL (36 pmol/L) | ECHO  European Society of cardiology* | | Test | LR | | --- | --- | | ECG | 1.6 | |  |
| Houghton et al [34]  1997  N=200 | 82.5% | Patients attending a HF clinic having been referred with suspected HF | Not reported | ECG  Abnormal if: HR <60 or > 100bpm, rhythm, cardiac axis, absent P waves, PR interval, QRS morphology, QRS duration, ST segment elevation/ depression, QT interval duration, T wave morphology, large U waves | ECHO  EF<40%, FS<25%, impaired global functioning. | | Test | LR | | --- | --- | | ECG | 1.6 | |  |
| Landray et al [35]  2000  N=126 | 31.7% | Patients suspected by their GP to have HF referred to hospital clinic | Not reported | ECHO  BNP  CXR  ECHO: not defined  BNP >17.9pg/mL (5.2pmol/L)  CXR: pulmonary oedema or cardiomegaly | ECG  Abnormal if: Q waves, BBB, T wave inversion or LVH present | | Test | LR | | --- | --- | | MI | 3.6 | | ECG | 3.1 | | CXR | 1.2 | | BNP>17.9pg/mL | 1.3 | | MI & ECG | 3.3 | | Additional diagnostic value of BNP is small.  BNP cutoff used is the lowest of any study in this systematic review. |
| Lim et al [36]  N=137 | 14% | Referred from primary care to confirm diagnosis of LVSD | Not reported | ECG- criteria not reported  NTproBNP threshold 169.14pg/mL (20pmol/L) | ECHO  Ejection fraction <45% | ECG LR+ 1.4  NTproBNP LR+ | Details limited as published as a letter |
| Lindsay et al [37]  2000  N=416 | 23% | Referrals to direct access ECHO service | Not reported | ECG  Abnormal ECG: presence of pathological Q-waves representing previous MI, ST-T changes, LAD, left atrial enlargement, LBBB, AF, evidence of heart block & poor R wave progression | ECHO  FS assessed via an M-mode through tips of mitral valve leaflets in long-axis parasternal view  If not possible then “eyeball” assessment of LV function made | |  | LR | | --- | --- | | Hx of MI | 3.9 | | ECG | 1.4 | | Combined | 1.5 | |  |
| Misuraca et al [38]  2002  N=83 | 54.2% | Patients referred by GP to ambulatory hospital with a diagnosis of CHF | Not reported | History & Examination  ECG  BNP  Abnormal ECG  BNP> 69.20pg/mL (20 pmol/L) | ECHO  EF ≤ 45% or EF > 45%, diastolic LV diameter <3.2 cm/m^2^ and abnormal transmitral flow | | Cutoff | LR | | --- | --- | | BNP> 69.20pg/mL (20 pmol/L) | 1.41 | | Paper translated from Italian |
| Nielsen et al [39]  2000  N=126 | 11.9% | Subset of patients from a cross-sectional study in primary care. Patients identified by review of case notes, prescription lists and by mailed questionnaire | |  | % | | --- | --- | | MI | 25% | | Angina | 29% | | History & Examination  ECG  CXR  N-ANP  Abnormal ECG  CXR  N-ANP 0.8nmol/l | ECHO  FS < 26% (equivalent to EF <45%)  Wall motion index score<1.5  LVSD | |  | LR | OR | | --- | --- | --- | | Previous MI | 2.1 | Not sig | | N-ANP | 3.9 | 5.0 | | HR | 4.1 | 9.0 | | Abnormal ECG | 2.0 | 18.0 | | Abnormal ECG, Heart rate>DBP, N-ANP all significantly associated on multivariate analysis |
| Shah et al [40]  2004  N=963 | 30.8% | Patients referred from primary care to HF clinic over 7-year period.  Patients either had symptom/signs - dyspnoea, fatigue & oedema; already managed as having HF but without definitive diagnosis; patients with factors associated with increased risk of developing HF | Not reported | Signs  ECG  CXR  Abnormal ECG: Q wave, ST segment, T wave abnormalities, LVH, LBBB, atrial hypertrophy, any rhythm disturbance  CXR: PA view with cardiothoracic ratio >0.5 | ECHO  LSVD ≡ EF <50% | Logistic regression analysis revealed 4 independent predictors of LVSD: Abnormal ECG; cardiomegaly on CXR; male sex; Hx of diabetes.   | No. of predictors | LR | | --- | --- | | 0 | 0.20 | | 1 | 0.37-0.71 | | 2 | 0.77-2.09 | | 3 | 2.53-5.0 | | 4 | 8.99 | | High pre-test probability based on heterogeneous reasons for referral.  Symptoms not elicited; only patient’s functional class using NYHA classification |
| Sim et al [41]  2003  N=83 | 31.3% | Breathless patients referred for ECHO from primary care | Not reported | BNP  BNP 19pg/mL (5.5pmol/L)  BNP 20pg/mL (5.8pmol/L) | ECHO  EF <35% | | BNP cutoffs | LR | | --- | --- | | 19pg/mL | 2.0 | | 20pg/mL | 2.3 | |  |
| Sparrow et al [42]  2003  N=621 | 50.6% | Patients in primary care taking a loop diuretic. 1366 identified, 1301 records viewed, 737 underwent full clinical assessment, ECG performed on 621 | |  | % | | --- | --- | | Dyspnoea | 67% | | Orthopnoea | 33% | | JVP | 11% | | MI | 27% | | Abnormal ECG | 65% | | Past MI & orthopnoea | 11% | | Past MI & any sign | 17% | | History & Examination  ECG  BNP  Abnormal ECG  BNP: 53.0pg/mL (15.3pmol/L) | ECHO  EF <40% | |  | LR | | --- | --- | | Dyspnoea | 1.2 | | Orthopnoea | 1.3 | | JVP | 2.6 | | MI | 1.4 | | Abnormal ECG | 1.3 | | BNP | 1.3 | | Past MI & orthopnoea | 1.8 | | Past MI & any sign | 1.7 | | Combined symptoms recorded by multivariable regression |
| Turley et al [43]  N=1426 | 17.5% | Retrospective study of two patient cohorts over 1 year of patients referred to two hospitals with suspected LVSD | Not reported | NTproBNP ≥150pg/mL | ECHO  “Quantitative overall assessment made” | NTproBNP LR+ | Details limited; short report |
| Yamamoto et al [44]  2000  N=466 | 10.9% | Patients referred for ECHO due to symptoms suggestive of HF | |  | % | | --- | --- | | CHF Symptoms | 33% | | Hypertension | 54% | | CAD | 26% | | MI | 10% | | Diabetes | 15% | | Clinical Score: ≥ 1 of  Hx MI, Previous HF Dx  Orthopnoea/PND  ECG: Presence of pathological Q waves or intraventicular conduction defect  CXR: Cardiomegaly, pulmonary venous hypertension. Interstitial oedema | ECHO  EF <45% | | Test | LR | | --- | --- | | Clinical Score | 2.5 | | BNP | 2.2 | |  |
| Zaphiriou et al [45]  2005  N=306 | 34% | Patients referred by GP with symptoms suggestive of HF | |  | **%** | | --- | --- | | Hypertension | 55% | | Diabetes | 19% | | MI | 14% | | ECG  BNP  NT-proBNP  ECG: Qualitative  BNP: 100, 65, 30 pg/mL  (28.9, 18.8, 8.7pmol/L)  NT-proBNP: 125, 166pg/mL  (14.75, 19.6pmol/L) | ECHO  European Society of cardiology* | |  | LR | | --- | --- | | ECG | 2.1 |  | BNP cutoff | LR | | --- | --- | | >100pg/mL | 2.8 | | >65pg/mL | 2.0 | | >30pg/mL | 1.5 |  | NT-proBNP | LR | | --- | --- | | >125pg/mL | 1.5 | | >166pg/mL | 1.7 | |  |

§ BNP: 1pg/mL = 0.289 pmol/L

NT-pro BNP: 1pg/mL = 0.118 pmol/L

**Abbreviations:**

AF = Atrial Fibrillation; ANP = Atrial Natriuretic Peptide; BNP = Brain Natriuretic Peptide; COPD = chronic obstructive pulmonary disease; CXR = Chest X-ray; DBP = Diastolic Blood Pressure; ECG = Electrocardiogram; ECHO = Echocardiogram; EF = Ejection Fraction; FS = Fractional Shortening; HF = Heart Failure; IHD = Ischaemic Heart Disease; LAD = Left Axis Deviation; LBBB = Left Bundle Branch Block; LVH = Left Ventricular Hypertrophy; LVSD = Left Ventricular Systolic Dysfunction; N-ANP = N-terminal proANP; N-BNP = N-terminal BNP; PND = Paroxysmal Nocturnal Dyspnoea; RAD = Right Axis Deviation;

* European Society of Cardiology definition: Symptoms (shortness of breath, fatigue, fluid retention or any combination), signs of fluid retention (pulmonary or peripheral) in the presence of an underlying abnormality of cardiac structure and function. If doubt remains beneficial response to therapy is also diagnostic criteria.
