# Supplementary material for: The accuracy of symptoms, signs and diagnostic tests in the diagnosis of left ventricular dysfunction in primary care: A diagnostic accuracy systematic review
Source: BMC Fam Pract. 2008 Oct 8;9:56. doi: 10.1186/1471-2296-9-56 (PMC2569936; doi:10.1186/1471-2296-9-56)
Supplement: Additional file 3 — Table 3. Methodological standards for studies. [file 1471-2296-9-56-S3.doc]

**Methodological standards for studies deriving a clinical decision rule for LVSD in primary care**

| Study ID | All important predictors included? | Were all important predictors present in a significant proportion of study population | Were all outcome events and predictors clearly defined? | Blind assessment of predictors in outcome assessors? | Adequate sample size? | Does rule make clinical sense? |
| --- | --- | --- | --- | --- | --- | --- |
| Alehagen et al^22^ | Symptoms: No  Signs: No  Diagnostic tests: NT-proBNP | Yes | Predictors: Yes  Outcome: Yes | Yes | Unclear | Yes |
| Aspromonte et al^23^ | Symptoms: No  Signs: No  Diagnostic tests: BNP | Unclear | Predictors: No  Outcomes: Yes | Yes | Unclear | Yes, BNP is cost effective for targeted screening |
| Atisha et al^24^ | Symptoms: No  Signs: No  Diagnostic tests: BNP | Yes | Predictors: Yes  Outcome: Yes | Yes | Unclear | Yes |
| Cowie et al^25^ | Symptoms: No  Signs: No  Diagnostic tests: ANP, BNP | Unclear | Predictors: Yes  Outcome: Yes | No | Unclear | Yes for diagnostic tests (BNP) |
| Davie et al^26^ | Symptoms: No  Signs: No  Diagnostic tests: Yes | Unclear | Predictors: No  Outcome: Yes | Yes | Unclear | Yes, LR+ of 2.4 with “major” ECG abnormality |
| Davie et al^27^ | Symptoms: Yes  Signs: Yes  Diagnostic tests: No | Yes | Predictors: Yes  Outcome: Yes | No | Unclear | Yes |

| Fahey et al^28^ | Symptoms: Yes  Signs: Yes  Diagnostic tests: ECG | Yes | Predictors: Yes  Outcome: Yes | Yes | Yes | Yes |
| --- | --- | --- | --- | --- | --- | --- |
| Fox et al^29^ | Symptoms: Partly  Signs: Partly  Diagnostic tests: ECG, CXR | Yes | Predictors: Yes  Outcome: Yes | No | Unclear | Yes but only for selected signs, symptoms, ECG & CXR |
| Fuat et al^30^ | Symptoms: No  Signs: No  Diagnostic tests: BNP, NT-proBNP | Unclear | Predictors: Yes  Outcome: Yes | Unclear | Unclear | Yes, although cutoff points have been identified they need “validation” |
| Gustafsson et al^31^ | Symptoms: No  Signs: No  Diagnostic tests: NT-proBNP | Yes | Predictors: Yes  Outcome: Yes | Unclear | Unclear | Yes |
| Hess et al^32^ | Symptoms: No  Signs: No  Diagnostic tests: BNP | Unclear | Predictors: Yes  Outcome: Yes | Yes | Unclear | Yes |
| Hobbs et al^33^ | Symptoms: No  Signs: No  Diagnostic tests: NT-proBNP | Yes | Predictors: No  Outcome: Yes | Unclear | Unclear | Yes |
| Houghton et al^34^ | Symptoms: No  Signs: No  Diagnostic tests: ECG | Yes | Predictors: Yes  Outcome: Yes | Unclear | Unclear | Yes (LR=1.6) |
| Landray et al^35^ | Symptoms: No  Signs: No  Diagnostic tests: Yes | Unclear | Predictors: No  Outcome: Yes | Unclear | Unclear | Yes, LRs similar to other “referral” studies |

| Lindsay et al^37^ | Symptoms: Yes  Signs: No  Diagnostic tests: ECG | Unclear | Predictors: Yes  Outcome: Yes | Yes | Unclear | Yes |
| --- | --- | --- | --- | --- | --- | --- |
| Lim et al^36^ | Symptoms: No  Signs: No  Diagnostic tests: ECG, NTproBNP | Unclear | Predictors: No  Outcome: Yes | Yes | Unclear | Yes in terms of diagnostic test |
| Misuraca et al^38^ | Symptoms: No  Signs: No  Diagnostic tests: ECG, BNP | Unclear | Predictors: Unclear  Outcome: Unclear | Unclear | Unclear | Yes (high NPV) |
| Nielsen et al^39^ | Symptoms: No  Signs: Yes  Diagnostic tests: CXR, ECG, N-ANP | Unclear | Predictors: Yes  Outcome: Yes | Yes (for both predictor, ECG assessment & outcome ECHO) | Unclear | Yes, but only for selected signs, ECG & N-ANP |
| Shah et al^40^ | Symptoms: No  Signs: Yes  Diagnostic tests: Yes | Unclear | Predictors: No  Outcome: Yes | Unclear | Unclear | Yes, but not all symptoms elicited & heterogeneous patient population |
| Sim et al^41^ | Symptoms: No  Signs: No  Diagnostic tests: BNP | Yes | Predictors: No  Outcome: Yes | Unclear | Unclear | Yes. Concludes that BNP is a cost effective pre-screening tool for ECHO |
| Sparrow et al^42^ | Symptoms: Yes  Signs: Yes  Diagnostic tests: Yes | Yes | Predictors: Yes  Outcome: Yes | Yes | Unclear | Clinical diagnosis is often inaccurate and that open access echocardiography is required |
| Turley et al^43^ | Symptoms: No  Signs: No  Diagnostic tests: Yes | Unclear | Predictors: No  Outcome: Yes | Unclear | Unclear | Yes but only as diagnostic test |
| Yamamoto et al^44^ | Symptoms: No  Signs: No  Diagnostic tests: BNP | Yes | Predictors: Yes  Outcome: Yes | Yes | Unclear | Yes |
| Zaphiriou et al^45^ | Symptoms: No  Signs: No  Diagnostic tests: BNP, NT-proBNP, ECG | Unclear | Predictors: No  Outcome: Yes | Yes | Unclear | Yes. Only for NT-proBNP |
