# Supplementary material for: The accuracy of symptoms, signs and diagnostic tests in the diagnosis of left ventricular dysfunction in primary care: A diagnostic accuracy systematic review
Source: BMC Fam Pract. 2008 Oct 8;9:56. doi: 10.1186/1471-2296-9-56 (PMC2569936; doi:10.1186/1471-2296-9-56)
Supplement: Additional file 4 — Table 4. Clinical values [file 1471-2296-9-56-S4.doc]

Clinical value of symptoms, signs and diagnostic tests for LVSD

| Diagnostic Test | No. of Studies | No.of Patients | Pooled PLR | (CI) or Range | I-squared | Pooled NLR | (CI) or Range | I-squared |
| --- | --- | --- | --- | --- | --- | --- | --- | --- |
| Clinical History |  |  |  |  |  |  |  |  |
| History of MI | 6 | 1946 | 2.86 | 1.37 - 4.40 | 85.8% | 0.69 | 0.48 - 0.89 | 77.5% |
| Diabetes | 2 | 717 | 2.29 | 0.86 - 6.65 | 84.4% | 0.95 | 0.89 - 1.02 | 59.1% |
| Hypertension | 2 | 717 | 0.58 | (0.39 - 0.87) | 0.0% | 1.30 | (1.13 - 1.50) | 0.0% |
| Male | 2 | 1471 | 1.61 | (1.41 - 1.84) | 0.0% | 0.68 | (0.60 - 0.77) | 0.0% |
| Symptoms |  |  |  |  |  |  |  |  |
| Fatigue | 2 | 1079 | 1.03 | (0.84 - 1.25) | 34.4% | 0.98 | 0.88 - 1.17 | 52.0% |
| Dyspnoea | 3 | 1338 | 1.15 | (1.09 - 1.21) | 4.8% | 0.50 | (0.20 - 1.26) | 45.8% |
| Orthopnoea | 3 | 1338 | 1.59 | 0.89 - 3.58 | 83.8% | 0.89 | 0.77 - 1.04 | 58.6% |
| PND | 3 | 1338 | 1.71 | 1.12 - 2.23 | 57.4% | 0.87 | 0.75 - 0.99 | 78.2% |
| Signs |  |  |  |  |  |  |  |  |
| Peripheral oedema | 4 | 1721 | 1.18 | 0.96 - 1.48 | 68.0% | 0.92 | 0.74 - 1.05 | 51.8% |
| Abnormal Breath Sounds | 4 | 1721 | 1.53 | (1.17 - 1.19) | 44.4% | 0.85 | 0.64 - 0.94 | 75.6% |
| Raised JVP | 3 | 1338 | 4.36 | 2.66 - 7.44 | 58.1% | 0.88 | (0.83 - 0.91) | 8.2% |
| Displaced Apex Beat | 2 | 583 | 15.96 | (8.24 - 30.93) | 0.0% | 0.58 | 0.35 - 0.93 | 98.5% |
| 3rd Heart Sound | 3 | 1326 | 7.34 | 1.56 - 32.37 | 87.3% | 0.92 | 0.77 - 0.96 | 71.4% |
| Tests |  |  |  |  |  |  |  |  |
| CXR | 2 | 1089 | 1.47 | 1.19 - 1.71 | 80.0% | 0.58 | 0.49 - 0.77 | 66.8% |
| ECG | 11 | 3570 | 2.13 | (1.95 - 2.33) | 47.6% | 0.27 | 0.06 - 0.76 | 89.7% |
| BNP (cut -offs nearest 15pmol/L) | 9 | 2251 | 1.90 | 1.26 – 6.20 | 88.8% | 0.30 | 0.02 - 0.80 | 88.3% |
| NT-proANP | 2 | 238 | 2.98 | (2.24 - 3.97) | 0.0% | 0.18 | 0.04 - 0.64 | 93.5% |
| NT-proBNP | 8 | 3494 | 1.39 | 1.09 - 1.80 | 91.3% | 0.26 | 0.02 - 0.80 | 83.1% |
| Combined |  |  |  |  |  |  |  |  |
| ECG and Hx of MI | 2 | 542 | 2.80 | (2.43 - 3.24) | 0.0% | 0.13 | 0.03 - 0.48 | 96.8% |
